# The influence of depth of anesthesia and blood pressure on muscle recorded motor evoked potentials in spinal surgery. A prospective observational study protocol

**DOI:** 10.1007/s10877-020-00645-1

**Published:** 2021-01-28

**Authors:** Sebastiaan E. Dulfer, M. M. Sahinovic, F. Lange, F. H. Wapstra, D. Postmus, A. R. E. Potgieser, C. Faber, R. J. M. Groen, A. R. Absalom, G. Drost

**Affiliations:** 1 Department of Neurosurgery, University Medical Center Groningen, University of Groningen, Groningen, The Netherlands; 2 Department of Anesthesiology, University Medical Center Groningen, University of Groningen, Groningen, The Netherlands; 3 Department of Neurology, University Medical Center Groningen, University of Groningen, Groningen, The Netherlands; 4 Department of Orthopedics, University Medical Center Groningen, University of Groningen, Groningen, The Netherlands; 5 Department of Epidemiology, University Medical Center Groningen, University of Groningen, Groningen, The Netherlands

**Keywords:** Intraoperative neurophysiological monitoring, Blood pressure, Depth of anesthesia, Motor evoked potentials

## Abstract

For high-risk spinal surgeries, intraoperative neurophysiological monitoring (IONM) is used to detect and prevent intraoperative neurological injury. The motor tracts are monitored by recording and analyzing muscle transcranial electrical stimulation motor evoked potentials (mTc-MEPs). A mTc-MEP amplitude decrease of 50–80% is the most common warning criterion for possible neurological injury. However, these warning criteria often result in false positive warnings. False positives may be caused by inadequate depth of anesthesia and blood pressure on mTc-MEP amplitudes. The aim of this paper is to validate the study protocol in which the goal is to investigate the effects of depth of anesthesia (part 1) and blood pressure (part 2) on mTc-MEPs. Per part, 25 patients will be included. In order to investigate the effects of depth of anesthesia, a processed electroencephalogram (pEEG) monitor will be used. At pEEG values of 30, 40 and 50, mTc-MEP measurements will be performed. To examine the effect of blood pressure on mTc-MEPs the mean arterial pressure will be elevated from 60 to 100 mmHg during which mTc-MEP measurements will be performed. We hypothesize that by understanding the effects of depth of anesthesia and blood pressure on mTc-MEPs, the mTc-MEP monitoring can be interpreted more reliably. This may contribute to fewer false positive warnings. By performing this study after induction and prior to incision, this protocol provides a unique opportunity to study the effects of depths of anesthesia and blood pressure on mTc-MEPs alone with as little confounders as possible.

**Trial registration number** NL7772.

## Background

For complex and high risk spinal surgeries, intraoperative neurophysiological monitoring (IONM) is used, with the aim of detecting and preventing intraoperative neurological injury [1]. For monitoring in spinal surgery, it is advised to monitor both sensory tracts, using somatosensory evoked potentials (SSEPs), and motor tracts, using muscle recorded transcranial electrical stimulation motor evoked potentials, mTc-MEPs [1–4].

The most common warning criterion for possible surgically induced structural neurological injury, is an mTc-MEP amplitude decrease of 50–80% from the baseline value [2, 4–8]. Therefore, an mTc-MEP amplitude decrease of 50–80% warrants immediate exploration of possible causal factors. However, these warning criteria often result in false positive warnings [2]. This can be due to technical issues, but also other factors may influence mTc-MEP amplitudes, including direct effects of anesthetic drugs on neuronal transmission, as well as indirect effects of blood pressure, blood oxygen levels and body temperature resulting in false positive decreases in mTc-MEPs [9–15]. In clinical practice, decreasing the concentration of intravenous anesthetic drugs and elevating blood pressure often help to restore the mTc-MEP amplitude.

It is known that for optimal recording of mTc-MEPs, total intravenous anesthesia (i.e. induction and maintenance of general anesthesia with intravenous infusions of propofol and an opioid) provides more reliable mTc-MEPs than volatile anesthesia (maintenance of anesthesia by administration of a volatile drug through the anesthetic breathing circuit) [16]. However, mTc-MEPs are still very sensitive to the suppressive effects of propofol anesthesia [10, 16–19]. Given the intra- and inter-individual variability in the relationship between blood and effect-site propofol concentrations, and actual clinical effect, it is scientifically important to study the relationship between depth of anesthesia (actual clinical effect) and mTc-MEP characteristics.

Currently, in clinical practice, depth of anesthesia is often objectively assessed by the use of processed electroencephalogram (pEEG) monitors [20]. pEEG monitors acquire the EEG signal recorded by electrodes placed on the forehead (and thus reflecting the electrical activity of the frontal lobe), and use a mathematical algorithm to analyze this signal and generate output [20]. For most pEEG systems, the output is indexed to a range of values from 0 to 100, where 0 indicates no detectable cortical electrical activity (i.e. maximum drug effect) and where 100 indicates a completely conscious patient (i.e. no drug effect measurable) [20]. For optimal anesthesia depth it is recommended that to maintain pEEG values between 40 and 60 [21].

It could be argued that a conventional IONM EEG montage would provide you with even more information about the depth of anesthesia and its effects on mTc-MEP monitoring. In this study protocol we deliberately choose to measure depth of anesthesia with a pEEG monitor instead of a conventional IONM EEG montage, as this is strongly recommended by international guidelines [22]. Even though there is some debate concerning the usefulness (validity) of depth of anaesthesia monitoring in detecting and preventing awareness, their role in hypnotic drug effect titration is well established [23, 24]. In this study, our goal is to standardize the intraoperative hypnotic drug effect. The only validated and clinically convenient way to achieve this is by using a widely available pEEG monitor.(e.g. BIS, entropy or qCON monitor) [25]. Even though a IONM EEG montage would add some useful information, there is currently no known method of using this information to guide anesthetic drug titration.

Another major factor influencing mTc-MEP is cerebral and spinal cord perfusion [26, 27]. Perfusion of any tissue is influenced by vascular resistance and blood pressure. At present there are no clinically available methods to directly assess cerebral and spinal cord perfusion. Indeed, besides global measurements of cardiac output, there are no clinically available methods of directly monitoring perfusion of any tissue. Instead, in clinical practice anesthesiologists use blood pressure measurements as an indirect surrogate of perfusion. Hypotension can occur after induction of anesthesia. It is generally recommended that hypotension should be managed by a combination of optimizing hypnotic and analgesic drug dose, and by optimizing intravascular volume status and vascular tone. The latter is done by administration of fluids and vasopressors such as noradrenaline. Noradrenaline increases blood pressure, but also increases vascular resistance and therefore might even decrease tissue perfusion [10, 28, 29]. It is currently unknown what the exact effects of vasopressor-induced elevation of blood pressure is on mTc-MEPs in humans.

We hypothesize that by understanding the effects of depth of anesthesia and blood pressure on mTc-MEPs, the mTc-MEP monitoring can be interpreted more reliably, resulting in fewer false positive findings. Therefore, the aim of this study protocol is to investigate the effects of depth of anesthesia and blood pressure on mTc-MEPs.

In addition to depth of anesthesia and blood pressure, mTc-MEP measurements are also influenced by other parameters including blood loss, pain and manipulation/movements of the patient. Therefore, in order to investigate the effects of different depths of anesthesia, defined by pEEG, and different blood pressures, defined by the mean arterial pressure (MAP), on mTc-MEP measurements with as few as possible confounding parameters, it should be investigated after anesthetic induction and before surgical incision.

The goal of this paper is to validate this study protocol. In a review by Dwan et al. it was shown that after comparing study protocols with the published results, discrepancies were often found in which at least one primary outcome was changed, introduced or omitted in 50% [30]. Moreover, statistically significant and positive outcomes are more likely to be published, introducing publication bias [31, 32]. Publishing research protocols therefore reduces publication bias, prevents selective publication and increases the transparency of research.

We think that, especially in the field of IONM, the research quality of studies has to be raised to a higher level. By publishing this study protocol we aim to contribute to this.

## Methods/design

### Study design

This research protocol describes two prospective observational studies that will be performed in two parts, in patients that undergo spinal surgery with mTc-MEP monitoring. Study measurements will be performed after anesthetic induction and prior to the surgical incision. In part 1 we will examine the effects of depth of anesthesia, quantified by pEEG, on mTc-MEP characteristics. In part 2 we will examine the effects of blood pressure and noradrenaline induced increases in blood pressure, on mTc-MEP characteristics.

Patients will either participate in part 1 or part 2.

### Study setting

This study will involve patients undergoing spinal surgery with IONM. Eligible patients will be informed about one of the two prospective observational studies at the outpatient clinics or per mail. The studies will be performed at the University Medical Center Groningen at the Neurosurgery, Neurology, Anesthesiology and Orthopedic Departments.

### Inclusion criteria

In order to be eligible to participate in this study, patients must meet all of the following criteria:

- ≥ 12 years.
- Demonstrated spinal pathology for which surgery with the use of mTc-MEP monitoring has been planned.
- Signed and dated informed consent document prior to any study-related procedures.

### Exclusion criteria

A potential subject who meets any of the following criteria will be excluded from participation in this study:

- Patient refusal.
- Existing motor weakness in the left or right tibialis anterior, gastrocnemius, or abductor hallucis muscles.
- History of epilepsy.
- Contra-indications to IONM such as presence of a pacemaker or implantable cardioverter-defribillator.
- Patients with history of stroke or cranial lesions, increased intracranial pressure, heart failure and long-standing hypertension.

### Study objectives and hypotheses

#### Part 1: depth of anesthesia

##### Primary objective

To determine the effect of depth of anesthesia, quantified by pEEG, on the characteristics of mTc-MEP measurements in spinal surgery.

###### Hypothesis

Lighter depth of anesthesia, defined by higher values of pEEG will:

- Reduce the threshold voltage required to evoke an mTc-MEP.
- Increase the mTc-MEP amplitude and the mTc-MEP area under the curve (AUC).

##### Secondary objective 1

To determine if a combination of pEEG and propofol concentration (estimated effect-site concentration and measured plasma concentrations) and/or actual MAP during mTc-MEP registrations, better enable prediction of mTc-MEP characteristics than pEEG alone.

###### Hypothesis

- pEEG values alone enable better prediction of mTc-MEP characteristics than propofol concentrations alone..
- pEEG alone is as good as a more complex model involving pEEG, propofol concentrations and MAP, at enabling prediction of mTc-MEP characteristics.

#### Part 2: Blood pressure

##### Primary objective

To determine the effects of increasing the MAP with a vasopressor infusion on the characteristics of mTc-MEP measurements in spinal surgery.

###### Hypothesis

A Higher MAP will:

- Reduce the threshold voltage required to evoke an mTc-MEP.
- Increase the mTc-MEP amplitude and the mTc-MEP AUC.

A range of alternative hypotheses will also be explored. These include:

- There is a biphasic relationship between MAP and mTc-MEP amplitude and AUC (with an initial improvement and a subsequent decrease at higher noradrenaline doses and MAP values).
- The MAP at which optimal mTc-MEP amplitudes and AUCs are found is age-dependent.

#### Secondary objective 1

To determine if a combination of MAP, with pEEG and/or propofol concentration (estimated effect-site concentration, or estimated or measured plasma concentrations) enables better prediction of mTc-MEP characteristics than the MAP on its own.

##### Hypothesis

- Within the specified range of pEEG (40–60), MAP has a greater effect on mTc-MEP characteristics than depth of anesthesia (pEEG).
- MAP is as good as a more complex model involving MAP, propofol concentrations and pEEG, at predicting mTc-MEP characteristics.

### Muscle recorded transcranial electrical stimulation motor evoked potentials

Intraoperative mTc-MEPs will be performed according to a standard procedure using a custom-made constant voltage stimulator (NIM-Eclipse E4 IONM system, Medtronic BV, The Netherlands). Transcranial electrical stimuli will be applied at locations Cpl1–Cpl2 (1 cm posterior and 1 cm lateral to C1 and C2), a modification of the international 10–20 system. Muscle motor evoked potentials will be recorded using surface electrodes (3M® ECG) on at least the left and right tibialis anterior (TA), the left and right gastrocnemius (GAS) and the left and right abductor hallucis (AH) muscles.

For each muscle recording site, an mTc-MEP threshold will be determined. The mTc-MEP threshold is defined as the voltage required to obtain an amplitude of at least 50 µV. Besides this, stimulation settings will be optimized to achieve supramaximal stimulation. A pulse train will consist of 5 square wave stimuli with either a pulse duration of 0.075 ms with an inter stimulus interval (ISI) of 1.5 ms or a pulse duration of 0.300 ms with an ISI of 3 ms. Depending on the height of amplitude and the amount of muscles that will be elicitable one of these two settings is chosen. If the pulse duration of 0.075 ms will be chosen the ISI will be optimized as well, ranging from 1 to 4 ms.

### Anesthetic management

All patients will be screened by an anesthesiologist before surgery. On arrival at the operating room, an IV cannula and an invasive arterial cannula will be inserted in the hand or forearm. Fluid administration will consist of a continuous IV infusion of crystalloid solution of 500 cm^3^/h. All patients will receive total intravenous anesthesia with propofol and remifentanil administered using target-controlled infusion (TCI) pumps. Muscle relaxants will only be given prior to intubation to avoid the negative effects of muscle relaxation on the muscle responses. To be able to study the effects of MAP and depth of anesthesia alone, parameters that might influence the mTc-MEP measurements will be kept stable and in between certain ranges:

- Propofol will only be administered per TCI pump (i.e. no manual boluses will be administered). The target effect-site concentrations will be selected by the responsible clinician on the basis of the pEEG values as per standard practice in our hospital.
- Remifentanil will be administered at effect site concentration of 4 ng/ml, using the Minto PKPD model [33, 34].
- Ketamine use will not be administered during the study.
- In part 1, vasopressor infusions will be used to maintain the MAP at 70–100 mmHg.
- In part 2, propofol infusion rates will be adjusted to maintain the pEEG between 40 and 60.
- The ventilation will be adjusted to maintain normocarbia (end-tidal CO_2_ 4–5.0 kPa; or PaCO_2_ 4.5–5.5 kPa) to avoid the confounding influences of excessive anesthesia and hypocarbia on mTc-MEPs.
- Core body temperature will be kept close to 37 Celsius, with the use of a routinely used forced air patient warming system.
- The EV1000 monitor (Edwards Lifesciences, USA) will collect data concerning cardiac output, pulse pressure variation and stroke volume variation. The latter will be kept below 12% during the study procedure.

Data of all abovementioned parameters will be continuously measured and the data will be collected. Other anesthetic considerations specifically for either part 1 or part 2 are explained below at the study procedures.

### Study procedures

This research protocol describes two prospective observational studies that will be performed in two parts. In part 1 we will examine the effects of depth of anesthesia, quantified by pEEG, on mTc-MEP characteristics. In part 2 we will examine the effects of blood pressure, quantified by the MAP, on mTc-MEP characteristics.

Patients that will undergo spinal surgery with the use of mTc-MEPs are eligible for these studies. Patients will either participate in part 1 or part 2. This will not be randomized.

#### Part 1: effect of depth of anesthesia on mTc-MEP characteristics in spinal surgery

**Fig. 1:**
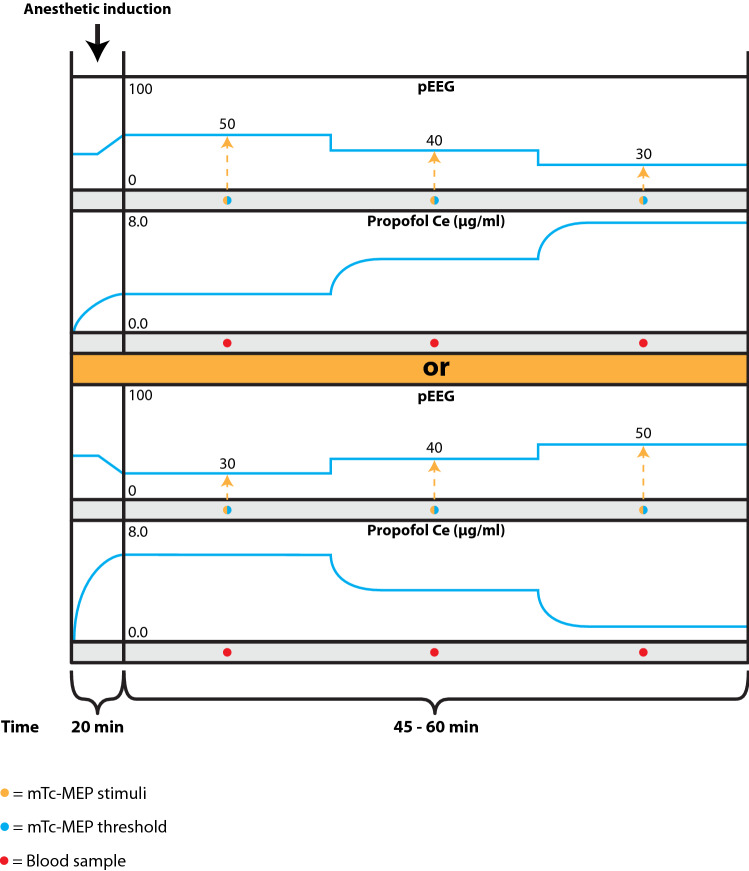
Schematic overview study design part 1: effects of depth of anesthesia on mTc-MEP characteristics in spinal surgery. *pEEG* processed electroencephalogram, *Propofol Ce* propofol effect site concentration, mTc-MEP muscle recorded transcranial electrical stimulation motor evoked potential

After induction of anesthesia and positioning the patient in prone position, and before the onset of the spinal surgery, the effect-site (brain) propofol concentrations will be adjusted to achieve pEEG values according to Fig. 1.

Although models exist to predict the time course of clinical effects of propofol through its interaction with the GABA_A_ receptor, there remains uncertainty over the accuracy of these models, and of the likelihood of longer-term structural changes following propofol interactions with the receptor. If there are long term effects, then path-dependent differences may exist. Patients will therefore be randomized over two different protocols. One group will start with decreasing propofol concentrations and one group will start with increasing propofol concentrations resulting into the pEEG values as can be observed in Fig. 1. These predefined pEEG values are not in conflict with the current standard care since pEEG ranging from 30 to 60 are common values during spinal surgery. Once a stable pEEG level has been reached, mTc-MEPs will be registered for 5 min and the following will be determined: a voltage threshold to evoke mTc-MEPs and three mTc-MEP measurements, for the left and right TA, GAS and AH muscles. We will perform three mTc-MEP measurements per stable pEEG level to address for the pre-existing variability between mTc-MEPs and average the measurements per stable pEEG level. There will be at least 1 min in between each mTc-MEP measurement to avoid facilitation.

A decrease of the hypnotic component of anesthesia without adequate analgesia (anti-nociception) could lead to increased nociception, increased sympathetic nervous system outflow and blood pressure rise [35]. In this study, however we shall suppress the effects of noxious mTc-MEP stimulation by administering remifentanil at an effect site concentration of 4 ng/ml, using the Minto PKPD model. Based on a previously published study by this research group [36] and the remifentanil package insert, this level of analgesia is enough to ensure adequate anesthesia (combined effect of hypnosis and analgesia) even when hypnotic drug effect is decreased to low hypnotic levels. This will limit the sympathetic outflow and ensure hemodynamic stability.

In order to determine if a combination of pEEG and propofol concentration (estimated effect-site concentration, or measured plasma concentrations) and/or actual MAP during mTc-MEP registrations, better predict mTc-MEP characteristics than the pEEG on its own, in total three blood samples will be taken (10 ml per blood sample) immediately after each of the series of mTc-MEP recordings to measure the propofol plasma concentrations. Propofol Ce values and MAP values will be collected directly after each mTc-MEP measurement as well. See Table 1 for the participant timeline.

**Table 1:** Participant timeline

		Before hospital admission	Day 1 of hospital admission	Day 2: procedure Before surgical incision	Day 2: procedure After surgical incision	Day 4/7
1	Approach, information provision	X				
2	Informed consent	X	X			
3	Inclusion in part 1 or 2	X	X			
4	Pre-operative neurological examination		X			
5	mTc-MEP measurements at different pEEG and MAP values			X		
6	Taking blood samples for propofol blood concentrations			X		
7	Collecting propofol Ce, MAP/pEEG values immediately after mTc-MEP measurements			X		
8	Post-operative neurological examination					X

*mTc-MEP* muscle recorded transcranial electrical stimulation motor evoked potential, *pEEG* processed electroencephalogram, *MAP* mean arterial pressure

#### Part 2: effect of blood pressure on mTc-MEP characteristics in spinal surgery

**Fig. 2:**
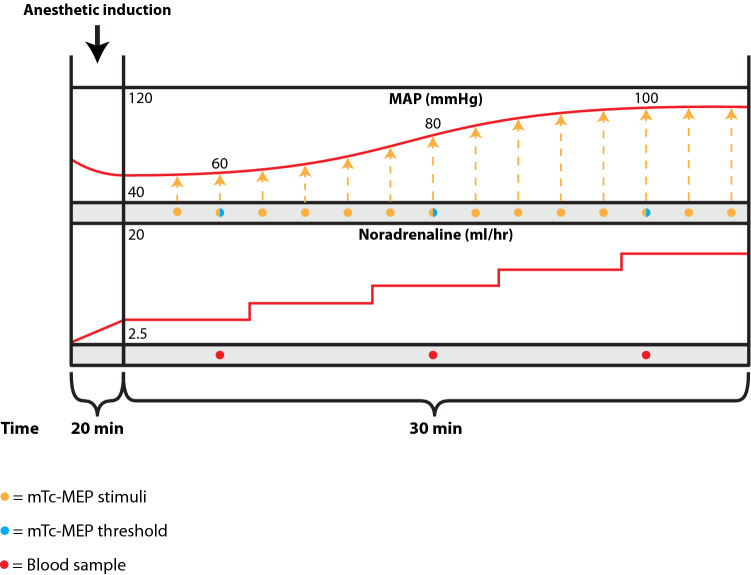
Schematic overview study design part 2: effects of blood pressure on mTc-MEP characteristics in spinal surgery. *MAP* mean arterial pressure, *mTc-MEP* muscle recorded transcranial electrical stimulation motor evoked potential

After induction of anesthesia and positioning the patient in prone position, and before the start of spinal surgery, the propofol infusion will be adjusted to achieve a target pEEG value of 40. After induction of anesthesia, to achieve a pEEG value of 40, the MAP is often 60 mmHg or lower. A slow intravenous infusion of noradrenaline will be started to slowly increase the MAP. Every 2 min, while increasing the infusion rates, mTc-MEPs will be performed for the left and right TA, GAS and AH hallucis muscles (Fig. 2). At MAP levels of 60 mmHg, 80 mmHg and 100 mmHg an mTc-MEP threshold will be measured for the abovementioned muscles. The noradrenaline infusion rate will be adjusted to achieve a gradual MAP increase over 30 min, until the MAP is approximately 100 mmHg. MAPs will be measured continuously using an invasive arterial cannula, which will have already been inserted for clinical care. In the event that the MAP after induction is greater than 60 mmHg but lower than 80 mmHg, we will accept that value, and use noradrenaline as necessary to increase the MAP to 100. In the event that the MAP is spontaneously > 100 mmHg immediately after induction, the patient will be withdrawn from the study, and further care passed on to the responsible anesthesiologist.

Due to the inadequacy of blood pressure to predict tissue perfusion directly, this study will also collect other calculated hemodynamic parameters generated by an EV1000 monitor consisting of cardiac output, pulse pressure variation and stroke volume variation.

In order to determine if a combination of MAP, with pEEG and/or propofol concentration (estimated effect-site concentration, or estimated or measured plasma concentrations) better predict mTc-MEP characteristics than the MAP on its own, in total three blood samples will be taken (10 ml per blood sample) at MAP values of approximately 60, 80 and 100 mmHg, to measure the propofol plasma concentrations. Propofol Ce values and pEEG values will be collected directly after each mTc-MEP measurement as well. See Table 1 for the participant timeline.

### Sample size calculation

#### Part 1:  pEEG

Very little is known about the quantitative relationship between depth of anesthesia, propofol plasma concentration, and mTc-MEP. There are insufficient data available on which to base a sample size calculation. A pragmatic sample size of 25 patients has therefore been chosen. This study should be regarded as a hypothesis-generating study.

#### Part 2: MAP

Although hypotension and hypoperfusion are well known causes of deterioration in mTc-MEP signals, and vasopressors are routinely used to increase the blood pressure when mTc-MEP signals deteriorate, very little is known about the quantitative relationship between vasopressor administration and mTc-MEP. Therefore, there is insufficient data available on which to base a sample size calculation. A pragmatic sample size of 25 patients has therefore been chosen. This study should be regarded as a hypothesis-generating study.

### Recruitment

The surgeon, neurophysiologist and anesthesiologist evaluate whether the patient is eligible for inclusion in one of these studies, following the above mentioned in- and exclusion criteria. Eligible patients will receive a written description of the study at their visit at the outpatient clinic or per mail. All candidates who have been sent the written information and consent form will be visited on the ward before their operation and asked if they have read and understood the information and whether they still have questions about the study. The researcher will ask the patient to sign the informed consent form and then co-sign the consent form.

### Data management

IONM data will be exported from the NIM-Eclipse E4 IONM system (Medtronic BV, The Netherlands) as csv file. After performing simple statistics using python, calculating the peak-peak amplitude and AUC, data will be recorded in Microsoft Access. Anesthesiology data and the neurological examination data will be recorded in a web-based data capture system (REDCAP).

### Statistical methods

For the statistical analyses of both the primary and secondary outcomes, a linear mixed effects model will be used to account for the repeated mTc-MEP measurements. Below, per outcome and per part of the study, the linear mixed effects models will be more precisely specified.

#### Part 1: pEEG

##### Primary outcome

For the primary outcome of part 1, a linear mixed effects model will be fitted in which pEEG target (categorical variable with values 30, 40 or 50) will be included as a fixed effect and a random intercept will be used to account for the between-subject variability in the mTc-MEP measurements. Independent normal distributions with mean zero and constant variance will be used to represent the within-subject errors.

##### Secondary outcome

For the secondary outcome, additional terms for MAP and propofol concentrations (effects site and blood concentration) will be added to the fixed effects structure of the model specified for the primary outcome. The specification of the random effects structure and the residual covariance structure will not change,

#### Part 2: MAP

##### Primary outcome

For the primary outcome of part 2, a linear mixed effects model will be fitted in which the MAP (continuous variable) will be included as a fixed effect and a random intercept as well as a random slope for MAP will be used to account for the between-subject variability in the mTc-MEP trajectories. An unstructured covariance matrix will be used for the covariance structure of the random effects. Independent normal distributions with mean zero and constant variance will be used to represent the within-subject errors.

##### Secondary outcome

For the secondary outcome, additional terms for pEEG and propofol concentrations (effects site and blood concentration) will be added to the fixed effects structure of the model specified for the primary outcome. The specification of the random effects structure and the residual covariance structure will not change.

#### Subgroup analysis

Due to the neurophysiological differences at different ages, a subgroup analysis to adjust for age may be performed if applicable.

### Data monitoring

Monitoring activities will be performed according to the Monitoring Plan. We will arrange 1 kick off meeting before the actual start of the study and 1 monitoring visit during the study. Monitoring will be performed in compliance with Good Clinical Practice and applicable national regulations.

Monitoring of the main case report form data will be done based on source data verification of a sample of case report forms (an estimated sample size of 10%). Main focus of the monitoring to be executed is related to the enrolment criteria, the informed consent procedure, safety parameters and the primary endpoint. Exact details will be documented in the monitor plan. Further activities will involve checks of the Site File, study specific procedures.

No close out visit will be performed. After the monitoring visit, the site will be instructed on how to prepare their documentation in such a way that it is ready for long-term archiving. The applicable documents will be archived over a period of 15 years.

### Harms

Due to the study protocol the total anesthesia time will be prolonged by approximately 30–60 min. This represents an increase in duration of the anesthesia of approximately 10%. Although the safety of anesthesia has improved substantially during the last decades, anesthesia is not risk free. While the risks of some complications may correlate with the duration of anesthesia, most published statistics describe the risk per episode of anesthesia. Currently, the published incidence of anesthesia related morbidity ranges from 0.06% for peripheral nerve injury to 1% for pulmonary complications while the incidence of anaesthesia related mortality ranges from 0.008 to 0.02% [37, 38]. Based on these statistics, the fact that our patients do not suffer from pre-existing cardio-respiratory problems, and the modest relative increase in anesthesia duration, we do not expect that prolongation of general anaesthesia, necessary for the execution of this study, will cause harm to our patients. We shall however regularly re-evaluate the study conduct to minimize the risk as far as possible.

## Discussion

The goal of this study is to investigate the effects of depth of anesthesia, defined by pEEG, and different blood pressures, defined by the mean arterial pressure (MAP), on mTc-MEP measurements. We hypothesize that by understanding the effects of depth of anesthesia and blood pressure on mTc-MEPs, the mTc-MEP monitoring can be interpreted more reliably. This may contribute to fewer false positive warnings.

While it is thought that excessively deep anesthesia may impair mTc-MEP signals, and thereby may cause a false positive decrease of an mTc-MEP amplitude, few studies have investigated the influence of anesthetic depth on mTc-MEPs [17, 29]. Some studies have investigated the effect of increasing the target propofol effect site concentration (Ce) on mTc-MEP amplitude and thresholds. They found that higher propofol Ce values were associated with lower mTc-MEP amplitudes and higher voltage thresholds [17, 18]. However, due to hysteresis, large inter-individual pharmacokinetic variability (causing inaccuracy in the pharmacokinetic models in the target controlled pumps) and pharmacodynamic variability, there can be a significant discrepancy between the propofol Ce and actual clinical effect [39]. Furthermore, the number of patients included in these studies was small, variability in the estimated propofol Ce was large and the relation between the mTc-MEP amplitude and the clinical hypnotic drug effect was not measured [17, 18].

Only a few studies have investigated the effects of blood pressure on mTc-MEP, most of which were performed in animal experimental models [12, 14, 15, 26, 27, 40–42]. Two studies showed that hypotension is associated with decreased mTc-MEP amplitude and increased voltage threshold [15, 43]. One of these studies showed that increasing the MAP above 85 mmHg the mTc-MEPs were restored to baseline in 20% (6/30) of the patients [15]. However, again the sample size was small. Moreover, the intervention of increasing the blood pressure was performed during surgery. This makes it difficult to interpret what other factors in this multifactorial process influenced the mTc-MEP amplitudes for both the patients in which the mTc-MEP amplitudes did restore as well for the patients in which the mTc-MEP amplitudes did not restore.

Therefore, since we will perform this study after anesthetic induction and prior to incision, this study protocol provides a unique opportunity to investigate the effects of depth of anesthesia and blood pressure on mTc-MEPs in patients undergoing spinal surgery with as little confounders as possible.

## Data Availability

Not applicable. Not applicable.

## Abbreviations

IONM: Intraoperative neurophysiological monitoring.
mTc-MEP: Muscle recorded transcranial electrical stimulation motor evoked potentials
pEEG: Processed electroencephalogram
MAP: Mean arterial pressure
SSEP: Somatosensory evoked potentials
Ce: Effect site concentration
AUC: Area under the curve
TA: Tibialis anterior
GAS: Gastrocnemius
AH: Abductor hallucis
